# Personalized Medication Response Prediction for Attention-Deficit Hyperactivity Disorder: Learning in the Model Space vs. Learning in the Data Space

**DOI:** 10.3389/fphys.2017.00199

**Published:** 2017-04-11

**Authors:** Hin K. Wong, Paul A. Tiffin, Michael J. Chappell, Thomas E. Nichols, Patrick R. Welsh, Orla M. Doyle, Boryana C. Lopez-Kolkovska, Sarah K. Inglis, David Coghill, Yuan Shen, Peter Tiño

**Affiliations:** ^1^Warwick Manufacturing Group, Institute of Digital Healthcare, University of WarwickCoventry, UK; ^2^Mental Health and Addiction Research Group, Department of Health Sciences, University of YorkYork, UK; ^3^School of Engineering, University of WarwickCoventry, UK; ^4^School of Psychology, Newcastle UniversityNewcastle upon Tyne, UK; ^5^Centre for Neuroimaging Sciences, King's College LondonLondon, UK; ^6^Division of Maternal and Child Health Sciences, Ninewells Hospital and Medical School, University of DundeeDundee, UK; ^7^Departments of Paediatrics and Psychiatry, University of MelbourneMelbourne, VIC, Australia; ^8^School of Computer Science, University of BirminghamBirmingham, UK

**Keywords:** attention-deficit hyperactivity disorder, Bayesian inference, machine learning, methylphenidate, mixed effects model, personalized medicine, prognosis, treatment response

## Abstract

Attention-Deficit Hyperactive Disorder (ADHD) is one of the most common mental health disorders amongst school-aged children with an estimated prevalence of 5% in the global population (American Psychiatric Association, 2013). Stimulants, particularly methylphenidate (MPH), are the first-line option in the treatment of ADHD (Reeves and Schweitzer, 2004; Dopheide and Pliszka, 2009) and are prescribed to an increasing number of children and adolescents in the US and the UK every year (Safer et al., 1996; McCarthy et al., 2009), though recent studies suggest that this is tailing off, e.g., Holden et al. (2013). Around 70% of children demonstrate a clinically significant treatment response to stimulant medication (Spencer et al., 1996; Schachter et al., 2001; Swanson et al., 2001; Barbaresi et al., 2006). However, it is unclear which patient characteristics may moderate treatment effectiveness. As such, most existing research has focused on investigating univariate or multivariate correlations between a set of patient characteristics and the treatment outcome, with respect to dosage of one or several types of medication. The results of such studies are often contradictory and inconclusive due to a combination of small sample sizes, low-quality data, or a lack of available information on covariates. In this paper, feature extraction techniques such as latent trait analysis were applied to reduce the dimension of on a large dataset of patient characteristics, including the responses to symptom-based questionnaires, developmental health factors, demographic variables such as age and gender, and socioeconomic factors such as parental income. We introduce a Bayesian modeling approach in a “learning in the model space” framework that combines existing knowledge in the literature on factors that may potentially affect treatment response, with constraints imposed by a treatment response model. The model is personalized such that the variability among subjects is accounted for by a set of subject-specific parameters. For remission classification, this approach compares favorably with conventional methods such as support vector machines and mixed effect models on a range of performance measures. For instance, the proposed approach achieved an area under receiver operator characteristic curve of 82–84%, compared to 75–77% obtained from conventional regression or machine learning (“learning in the data space”) methods.

## 1. Introduction

The ability to predict treatment response (or non-response) in patients with mental health issues is potentially beneficial to both clinicians and patients in a number of ways. First, any treatment is accompanied by the risk of adverse effects—where non-response is a probable outcome then the risks of treatment may outweigh the benefits. Second, prediction of treatment response may guide both the dose and choice of medication. For example, where adverse events are dose-dependent then a clinician may chose to abandon a treatment course if a patient was a probable non-responder. Third, response prediction helps to calibrate both clinician and patient expectations of treatment outcomes. Finally, identifying non-responders may prompt a re-appraisal of the diagnosis and formulation of a patient's problem—misdiagnosis being one potential cause of non-response. These benefits certainly apply to Attention-Deficit Hyperactive Disorder (ADHD), which is one of the most common developmental disorders among school-aged children with an estimated prevalence of 5% in the general population worldwide (American Psychiatric Association, 2013). Stimulants, particularly methylphenidate (MPH), are the first-line option in the treatment of ADHD (Reeves and Schweitzer, 2004; Dopheide and Pliszka, 2009). Stimulants are prescribed to an increasing number of children and adolescents in the US and the UK every year (Safer et al., 1996; McCarthy et al., 2009), though recent studies suggest that this trend is tailing off e.g., Holden et al. (2013). The beneficial effects of stimulant medication on the core symptoms of ADHD have been demonstrated by numerous clinical trials, reviews and meta-analyses (Banaschewski et al., 2006; Greenhill et al., 2006; van der Oord et al., 2008; Storebø et al., 2015). Nevertheless, adverse effects of the medications are also common (Storebø et al., 2015). The findings from previous research suggest that around 70% of children demonstrate a clinically significant treatment response to stimulant medication (Spencer et al., 1996; Schachter et al., 2001; Swanson et al., 2001; Barbaresi et al., 2006). However, it is unclear which patient characteristics may moderate treatment effectiveness and whether non-response can be predicted.

To date, achieving accurate predictions of the clinical outcomes for patients with ADHD has proven elusive—most of the literature has focused on investigating the potential correlations between a set of patient characteristics and the outcome following treatment with one or more types of medication. Information relating to patient characteristics has mostly been in the form of subjective questionnaire ratings, clinical notes and qualitative psychometric data; for example, the ratings from symptom-based questionnaires such as the Swanson, Nolan, and Pelham (SNAP) questionnaire (Swanson et al., 1983; Atkins et al., 1985; Swanson, 1992; Bussing et al., 2008), along with demographic variables such as age, sex and social economic background. The results from such studies are often contradictory and inconclusive due to small sample sizes and/or limited availability and quality of data, especially in the temporal (longitudinal) domain.

Along with more conventional statistical approaches, machine learning has also shown promise in predicting treatment response or prognosis in healthcare applications. Indeed, recently a random forest regression analysis was used to predict outcome in a group of patients affected by Obsessive Compulsive Disorder (OCD) from a relatively small pool of questionnaire items, with a reported error rate of 24.6% (Askland et al., 2015). Likewise, there has been a previous attempt to use machine learning techniques to predict treatment response in ADHD (Kim et al., 2015); support vector machine classification from this study was reported as 84.6% accurate (not to be confused with the balanced accuracy measure used in this paper). However, in addition to demographic and clinical questionnaire-derived data, the study used genetic as well as neuroimaging and neuropsychological information as inputs. Such data are unlikely to be readily available to clinicians in routine practice.

In this paper we investigate whether the inclusion of prior knowledge relating to the potential mechanism behind the presentation of a mental health condition and characteristics of individual patients can add value in predicting treatment. Thus, we hypothesized that a pragmatic machine learning approach based on a mechanistic or parametric model (a “learning in the model space” framework) for treatment response prediction may offer an advantage over more conventional methods (Brodersen et al., 2011; Doyle et al., 2013; Chen et al., 2014; Shen et al., 2016). This method represents each newly observed patient through a model; the models are personalized such that individual differences are accounted for by a set of subject-specific parameters. In the case of ADHD, developing a plausible mechanistic model is not straightforward—despite decades of research, the underlying mechanism for the disorder is not well understood. In addition, any mechanistic model would have to be based on data that are likely to be available in good, but routine, clinical practice.

This paper documents, within the “learning in the model space” framework, a Bayesian linear regression model for the prediction of treatment response in a cohort of children diagnosed and treated for ADHD in the UK. The performance of this new approach is then compared with conventional regression and machine learning methods (“learning in the data space”) to assess whether or not the new approach offers benefits, and if so under what circumstances.

## 2. Materials and methods

### 2.1. Participants

The children enrolled in the study were drawn from the ADHD Drug Use and Chronic Effects (ADDUCE) cohort study (The ADDUCE Consortium, 2016), covered by a data sharing agreement with patient consent. The participants were from the UK NHS Tayside region who had attended the ADHD treatment clinics held at Dundee and Perth, UK. 262 families of eligible children were contacted, of which 181 (70%) were recruited and data on 173 of them were obtained for the purpose of this study. In addition, data were available on 94 healthy controls. Out of the 173 patients (whose baseline data are available), 157 of them started dose optimization studies and therefore longitudinal (temporal) data are available (See Section 3.1). To be eligible for the ADHD group, children had to be 6–17 years of age, have a clinical diagnosis of ADHD (see below), have had no previous medical history of methylphenidate use (medication-naïve) and have parental and child consent/assent to commence. The criteria for the healthy control group were similar apart from them having no current or previous psychiatric diagnoses. The recruitment was carried out over a 30-month period from January 2012–August 2014.

All patients in the ADHD group had already been clinically diagnosed with ADHD; this diagnosis was based on the clinical judgment of the assessing physician, informed by structured interviews with parents/carers, information provided by the child's school, direct observation of the child at the clinic, and at times, in their educational setting. Thus, the physician had to be satisfied that the child fulfilled the diagnostic criteria for a hyperkinetic disorder according to the International Classification of Diseases 10th edition (ICD-10) (World Health Organization, 2010), or ADHD as defined by the Diagnostic and Statistical Manual 4th edition (DSM-IV) (American Psychiatric Association, 2000). This means that the child had to demonstrate disabling and pervasive inattentiveness, hyperactivity, and impulsivity across a range of settings. The clinic was designed to implement a “dose optimization titration” scheme of medication in children diagnosed with ADHD. This involved giving increasing doses of methylphenidate (as the first line medication) at roughly weekly intervals until remission from symptoms was achieved or problematic adverse effects were encountered. If remission was not achieved with a first line medication within recommended dosage limits, or if problematic side-effects were encountered then a second line drug was initiated, and again, increased in dosage, as before.

### 2.2. Assessment

A range of baseline social and demographic factors was recorded, including parental marital status, family composition, and socioeconomic status as indicated by the Scottish Index of Multiple Deprivation (SIMD) 2012 (APS Group Scotland, 2012) derived from the family home postcode. A history of any previous psychiatric or non-psychiatric medication exposure was recorded, as were any physical health issues. Verbal and non-verbal intellectual functioning was estimated from parental reports and any educational issues noted. Problems with anxiety and low mood were rated using the short form of the Mood and Feelings Questionnaire (MFQ) with the parents, and where appropriate, the child as informants (Angold et al., 1996). Dystonia and abnormal movements were recorded using the Abnormal Involuntary Movement Scale (AIMS) (Guy, 1974, pp. 534–537). Oppositional and ADHD symptoms and behaviors were rated, according to parental report, using the Swanson, Nolan, and Pelham (SNAP-IV) questionnaire (Swanson et al., 1983). Any substance used by the participants was recorded using the Substance Use Questionnaire (SUQ). Fine motor issues were recorded using the Developmental Coordination Disorder Questionnaire 2007 (DCDQ'07). Several sections of the Development and Well-Being Assessment (DAWBA) were used (Goodman et al., 2000); these were (1) Rapidly Changing Mood (child and parent versions), (2) Tic disorders, including the Tourette syndrome, (3) Awkward and troublesome behavior. Tic severity (where present) was also rated using the Yale Global Tic Severity Scale (YGTSS) (Leckman et al., 1989). Possible behaviors associated with an underlying Autism Spectrum Disorder (ASD) were evaluated using the Social Communication Questionnaire (SCQ) (Rutter et al., 2003). The Strengths and Difficulties Questionnaire (SDQ) (Goodman, 1997-07) was used to rate parental perceived levels of pro-social behavior, hyperactivity/impulsivity, conduct problems, emotional symptoms and peer relationship problems. The overall clinical impression was recorded using the Clinical Global Impression—Severity scale (CGI-S) (Guy, 1974, pp. 218–222) and Children's Global Assessment Scale (CGAS) (Shaffer et al., 1983).

Responses to medication, in terms of levels of ADHD symptoms, were reported by parents and recorded using the SNAP-IV questionnaire at each visit. Likewise, any potential adverse effects and co-morbidity problems were reported using the standard clinic proforma, along with weight, height and blood pressure of the child at each visit.

### 2.3. Feature extraction/factor analysis

The aforementioned questionnaires included a large number of items with categorical (binary or ordinal) response formats. Thus, in order to facilitate model development by reducing the dimensionality of the data whilst minimizing the loss of information, a series of factor (latent trait) analyses were conducted.

The key questionnaires used in the modeling process were the SCQ, the SDQ, and the SNAP-IV (see the previous section). In particular, the SNAP-IV scores served as the outcome variables, which indicated whether symptomatic remission had been achieved, following the dose-optimized titration of medication. The factor analyses sought to identify the dimensionality underlying the responses to the questionnaires and, consequently, the standardized factor scores represented the level of trait for each patient in that underlying dimension or construct.

In order to estimate the dimensionality, the sample of 173 patients and 94 healthy controls was randomly divided into two roughly equal exploratory and confirmatory datasets. A parallel analysis (Horn, 1965), adapted for categorical data, was then implemented in the freeware FACTOR (Lorenzo-Seva and Ferrando, 2006) using unweighted least squares (ULS) estimation method. A weighted “promax” rotation was deployed to achieve factor simplicity (Abdi, 2003). The maximum number of plausible factors (latent variables) was assumed to be indicated at the point where the eigenvalues of the factors in randomly generated data exceeded those observed in the real data. A series of exploratory factor analyses (EFAs–adapted for categorical dependent variables) were then conducted to aid interpretation of the factors. Oblique “geomin” rotation was used (Asparouhov and Muthén, 2009), assuming that, as in almost all psychological measures, underlying latent traits would be correlated with each other to some extent (Thurstone, 1931). A series of confirmatory factor analyses (CFAs) were then conducted using the held-back, confirmatory data (see Section 3.1 on cross-validation), in order to ensure that the factor structures derived fitted the data adequately. All EFAs and CFAs were conducted in the Mplus software version 7.1, using robust weighted least squares with mean and variance adjustment (WLSMV) as the estimation method (Muthén et al., 1997). Remission was defined by a child having a reported factor score in the hyperactive and inattentive dimensions (both elicited from factor analysis) equivalent to a mean item score in the SNAP-IV of one or less, which is conventionally taken to indicate symptomatic remission (Hechtman, 2005; Chou et al., 2012). The resulting symptom score thresholds are only slightly different for inattentiveness and hyperactivity (−0.97 vs. −0.92).

## 3. Modeling approach

**Figure 1:**
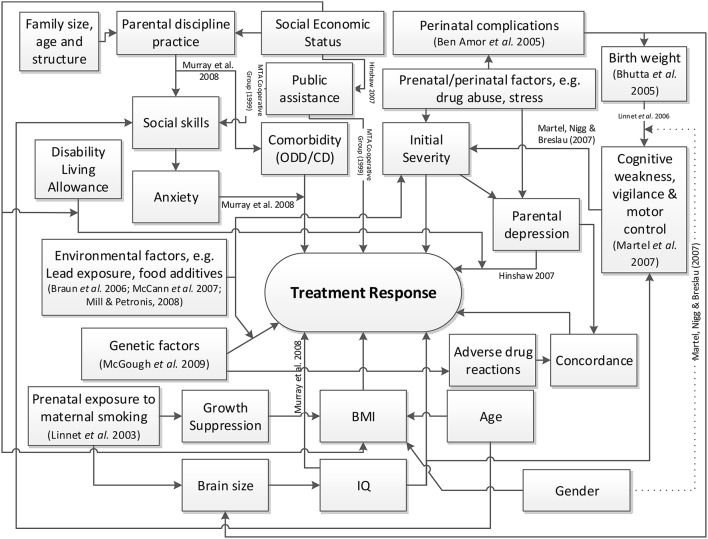
**High level causal factor model of treatment response prediction in ADHD**.

The causal factor model, shown in Figure 1, was derived using a rapid review approach to appraise and synthesize the existing evidence (Khangura et al., 2012). This model also took into account the nature of the data available in the cohort and was modified accordingly. The goal is not for the causal model to be comprehensive or definitive, but to identify from the literature as many potential factors relating to treatment response as there are available from the dataset, as well as helping to elicit the Bayesian prior distributions (Section 3.2.1). Model development was based on a literature review. This involved running searches in the EMBASE, MEDLINE and PsycINFO databases using the synonyms for ADHD (e.g., hyperkinesis) combined with terms relating to treatment outcome or response, and the names of the medications (both scientific and trademarks, full and abbreviated) prescribed in the cohort. The medications include 1) immediate release methylphenidate (IR-MPH, e.g., Ritalin®), 2) long-acting methylphenidate (XR-MPH, e.g., Concerta XL®, Equasym XL®, Medikinet XL®), 3) dextroamphetamine (DEX, e.g., Dexedrine®) including its prodrug lisdexamfetamine dimesylate (e.g., Elvanse®), and 4) atomoxetine (ATOM, e.g., Strattera®). Secondary sources were followed up. The quality of trial-based studies could be appraised using the CONSORT checklist (Schulz et al., 2010) and observational studies via the STROBE guidance (von Elm et al., 2007). Two of the authors (HKW and PAT) then made a judgment, based on the findings reported in the literature and the perceived likelihood of bias or uncertainty as to what extent variables in the model might be related to treatment response in ADHD. The model derived was consequently used to populate prior distributions for the patient-specific model parameters (i.e., the hyperpriors). Where the evidence was uncertain or inconsistent, the variances (i.e., imprecision) of the hyperpriors were increased.

**Figure 2:**
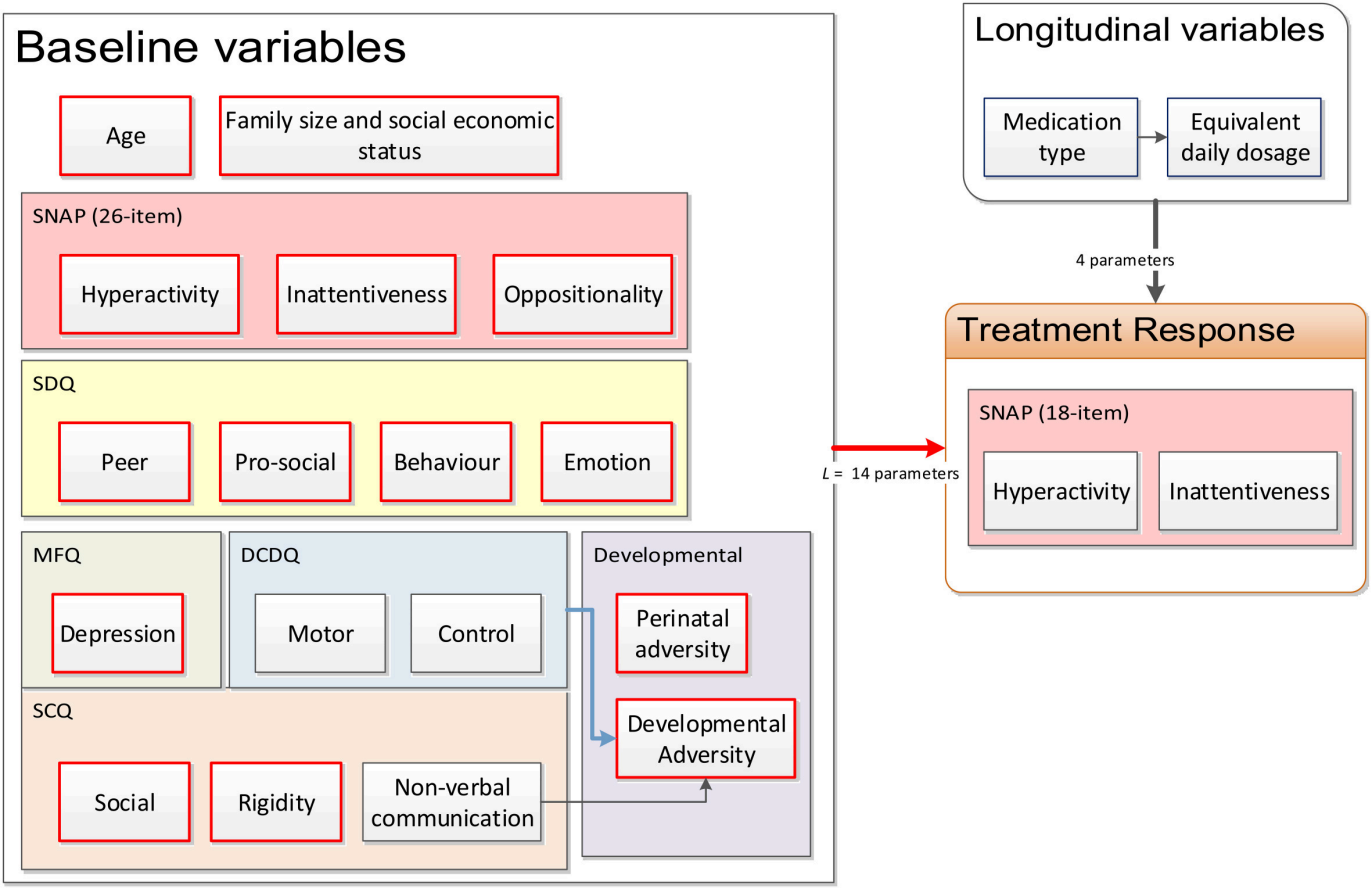
**Reduced causal factor model of treatment response prediction in ADHD**.

Not every piece of information mentioned in Section 2.2 was used for the purpose of modeling, because of insufficient data or multicollinearity between the variables. The causal factor model was then simplified based on the breadth of available data from the cohort, leading to a much reduced model as shown in Figure 2. Some factors were combined through another layer of feature extraction; for example, the *motor* and *control* latent factors, themselves also obtained from applying feature extraction to the DCDQ'07 questionnaire data (see Section 2.2), were combined with the *non-verbal communication* factor from the SCQ questionnaire to obtain a developmental adversity factor. Some factors were not obtained from standard questionnaires; for example, the *perinatal adversity* factor (see Figure 2) was constructed from birth weight and gestation age; the *family size and socioeconomic status* factor combined the number of siblings, parental house ownership (owned, mortgaged or rented) and the SIMD 2012 index (APS Group Scotland, 2012).

### 3.1. Data

There were 267 subjects whose baseline characteristics were measured (173 clinically diagnosed with ADHD and 94 healthy controls) at the first clinical appointment. Of the 173 non-controls, 157 were enrolled in dose optimization titration studies with parental consent, for whom longitudinal (temporal) data are available. The 157 patients with longitudinal data were randomized and 10-fold cross-validation partitions were constructed. Subjects were partitioned into 10 subgroups of roughly equal size in a patient-coherent fashion, i.e., data from a single patient only appeared in a single fold.

For all models investigated in this paper, a single fold was used as the validation dataset and the remaining nine folds were combined to serve as the training dataset. This process was iterated until each fold had served as validation data exactly once.

#### 3.1.1. Baseline characteristics

We labeled the patient subjects by the indexing variable *s* = 1, 2, …, *N*. A set of *L* patient-specific baseline continuous latent factors, encoded in a row vector **b**_*s*_ ∈ ℝ_1×*L*_ was obtained by performing feature extraction as described in Section 2.3 over the questionnaires detailed in Section 2.2. Referring to Figure 2, *L* = 14 factors were used for the baseline. Data from the controls in addition to the training dataset were utilized during feature extraction to ensure that the resulting latent factor models can sufficiently encompass the entire range of characteristics from ADHD patients to normal children. The resulting continuous latent factors would, in theory, be sufficiently representative of the information conveyed by the categorical questionnaire response variables.

To ensure that validation data were strictly not used for the model building, feature extraction was first performed using only training data from each of the folds (plus all the controls). This resulted in 10 sets of factor scores corresponding to each fold. The factor model structures (e.g., the number of factors per questionnaire) over the folds did not change across the folds, as statistical fit indices and Chi-square difference tests did not suggest that any changes were necessary. The factor models were then used to estimate the baseline factor scores for the validation sets in each of the folds.

Each of the 10 cross-validation runs resulted in a set of corresponding continuous latent factors, which were used as inputs to subsequent models. The models were trained and validated using the same training-validation partitioning used in the feature extraction process.

#### 3.1.2. Longitudinal data

Each of the 157 subjects with longitudinal data visited the clinic a varying number of times—from titration, stabilization to continuing care; the number of doctor's appointments, *A*_*s*_, varies from 1 to 22. At each appointment, the parent or guardian of the patient was asked to fill in an 18-item SNAP-IV questionnaire, which measures the degree of inattentiveness and hyperactivity. The responses were entered into a factor model (identified through feature extraction) to extract a continuous symptom score for inattentiveness and hyperactivity. We denote the appointment number by the indexing variable *a* so that *a* = 1, 2, …, *A*_*s*_. Let the independent “input” variables *m*_*a*, 1_, *m*_*a*, 2_, *m*_*a*, 3_, *m*_*a*, 4_ be the four types of medications, respectively, IR-MPH, XR-MPH, DEX, and ATOM for subject *s* at appointment *a*. Using datasheets for the medicines used, the dosages of DEX and ATOM were normalized to an equivalent daily dosage (EDD) of IR-MPH. For all *a* and *s*, this results in input and output matrices of the form:

(1) $$\begin{array}{rc} \text{Input}:{\text{M}}_{s} & =\left[\begin{array}{cccc} m_{1,1} & m_{1,2} & m_{1,3} & m_{1,4}\\ m_{2,1} & m_{2,2} & m_{2,3} & m_{2,4}\\ \vdots & \vdots & \vdots & \vdots \\ m_{a,1} & m_{a,2} & m_{a,3} & m_{a,4}\\ \vdots & \vdots & \vdots & \vdots \\ m_{A_{s},1} & m_{A_{s},2} & m_{A_{s},3} & m_{A_{s},4} \end{array}\right] \end{array}$$

(2) $$\begin{array}{rc} \text{Output}:{\text{y}}_{s} & ={\left[\begin{array}{ccccc} r_{1} & r_{2} & r_{3} & \ldots & r_{A_{s}} \end{array}\right]}^{⊺}, \end{array}$$

where *r* is the symptom severity measure and can be either the inattentiveness factor score or the hyperactivity factor score.

**Figure 3:**
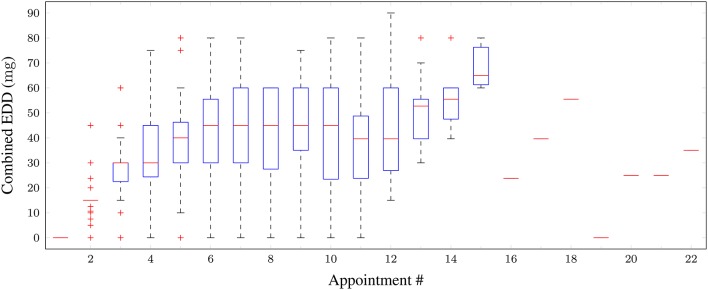
**Boxplot of combined equivalent (in IR-MPHunits) daily dosages of medications taken for all patients vs. appointment number**. Red horizontal lines: median; boxes: interquartile range; whiskers: 95% confidence intervals; red crosses: outliers.

The combined EDDs of medications (for the 4 types) prescribed over the appointment number for all patients are plotted as a boxplot in Figure 3. One can observe that as forced titration progressed over the appointments, the overall dosage level increased.

**Figure 4:**
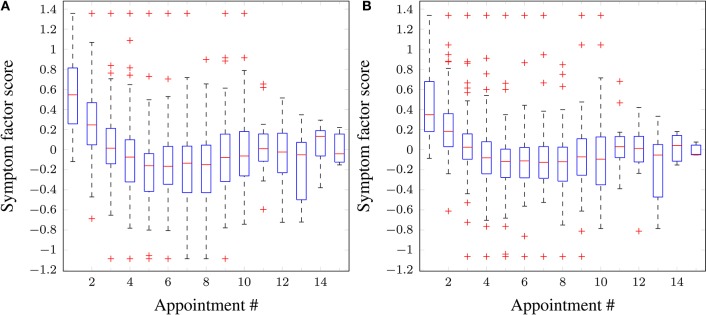
**Boxplots of symptom scores across all patients vs. appointment number**. **(A)** Inattentiveness, **(B)** hyperactivity. Red horizontal lines: median; boxes: interquartile range; whiskers: 95% confidence intervals; red crosses: outliers.

Figure 4 shows the distribution of inattentiveness and hyperactivity symptom factor scores for the patients for each appointment. The lower the factor scores, the less severe the symptoms are. In terms of a general trend, one can clearly see an effective and quick reduction in symptom levels over the first 5 appointments, as stimulant medication prescription ramps up during forced titration. The symptom scores cease to improve for appointments 6–8, after which a slight increase can be observed. This hints at adherence or persistence issues, but the available data do not allow further investigation—as such issues are not consistently reported by the parent/guardian or recorded in the clinical notes. While the model has no mechanism for modeling such effects, the adaptive learning nature of the Bayesian algorithm is able to self-correct and compensate for small deviations, for example, by “learning” to weight down the dose-response parameter for a given medication when the patient has a low adherence.

### 3.2. Treatment response model formulation

The treatment outcome is modeled as a linear combination of the baseline variables and the medication dosage,

(3) $$\begin{array}{r} {\text{y}}_{s}={\text{X}}_{s}\omega_{s}+\epsilon \end{array}$$

where

(4) Xs=[bs 1⋮Ms⋮bs 1],

$\epsilon \sim N\left(0,{\sigma_{\epsilon },s}^{2}\right)\in {\mathbb{R}}_{A_{s}\times 1}$ is an error term and ω_*s*_ ∈ ℝ_*P*×1_ is the *subject-specific* parameter vector moderating the effect of the baseline variables **b**_*s*_ on the treatment response, i.e., it accounts for how large an effect each of the various baseline variables or medication types has on treatment outcome. “Subject-specific” means that the parameter vector was allowed to be different for each subject so that patients with similar baseline characteristics can still have a different prediction outcome. The number of free parameters required is *P* = *L* + 4 + 1 = 19 for *L* = 14 (see Figure 2).

The baseline variables remain unchanged over different appointments while the medication dosage may vary according to the titration regime specified by the clinician. Hence, every row of the matrix **X**_*s*_ contains the same baseline characteristic vector for an individual patient, combined with the medication dosage vector. The row number in **X**_*s*_ corresponds to the appointment number.

Because the number of visits *A*_*s*_ of the subjects was usually fewer than the dimension of the parameter space *P*, the problem is mathematically underdetermined. Hence, classical least squares regression methods would fail without an appropriate regularization (Goodfellow et al., 2016). To this end, we employ a Bayesian formulation. In particular, the Bayesian linear regression was used to model the temporal evolution of the dose-response relationship for each patient.

In essence, a Bayesian approach allows prior or expert knowledge to be encoded into the problem formulation and this enables a probabilistic solution to be found despite the limited data available.

#### 3.2.1. Prior distributions and knowledge

The joint prior probability density function Pr(ω, σ^2^) is given in Equation (A3, Supplementary Material). In this exercise, the causal treatment response model based on the literature was used to constrain the prior of ω and its covariance matrix $\Lambda_{0}^{-1}$.

When eliciting the prior, quantitative information from the literature was not used, e.g., setting the mean of the prior distribution of the parameter vector omega to a specific numerical value. This is because the demography, sample sizes and effect sizes across literature vary and there is no correct way to normalize them. Instead, only the sign (direction) of the effect was encoded. For example, there is evidence that methylphenidate improves treatment response in the literature (a positive dose results in lower symptom score), therefore a negative value of −1 was specified for the columns of τ_0_ corresponding to *m*_*a*, 1_…*m*_*a*, 4_ in Equation (1). For positive associations with symptom scores, +1 was used instead. The same magnitude is used in other factors (i.e., either +1 or −1).

Each cohort study or clinical trial from the rapid review was appraised, respectively, using the STROBE and the CONSORT checklists by counting the number of pass and fail items out of the total. Evidence from the literature was marked as good quality when both the checklist score was similar to other studies on the same topic (within 20% from the best) and effect sizes were statistically significant as reported by the authors for the sample size used. The diagonals of the covariance matrix $\Lambda_{0}^{-1}$ were assigned an initial value of one; for every contradicting evidence (the effect sizes are opposite in direction) satisfying these criteria, 0.5 was added to the corresponding variance in the covariance matrix. A higher value may be specified if necessary, to ensure that the prior distribution of parameter omega spans both positive and negative sides sufficiently—within one standard deviation of τ_0_. On the other hand, if the effect sizes are positive the variance was reduced by 0.1 for each supporting studies, at the same time ensuring the variance does not go below 0.5. The (lack of) proposed existence of causal links between the variables in the causal model (see Figure 1) ensures the sparsity of the covariance matrix $\Lambda_{0}^{-1}$.

While these numbers may not be completely objective, the amount of data available means that the sensitivity of the results to the prior is low—sensitivity analysis shows that the effect of scaling the prior covariance between 50 and 150% of its original values changes the errors by about 5% of the training root mean squared (rms) error, and 3% for the validation rms error.

#### 3.2.2. Posterior distributions

The posterior distribution is given in Equation (A4, Supplementary Material), where the parameters of the distributions are obtained through Equation (A5) in Supplementary Material. In the training set, Bayesian learning uses data from all of the appointments that a subject had, in which case *n* = *A*_*s*_ where, as before, *A*_*s*_ is the total number of visits or appointments a subject has and data are available for. We introduce the simplified notations after Bayesian update has been applied to the training set using Equation (A5) in Supplementary Material, so that

(5) $$\begin{array}{r} \Lambda_{A_{s}}^{-1}\to {\hat{\Lambda }}_{s}^{-1},\;\tau_{A_{s}}\to \hat{\tau_{s}},\;\alpha_{A_{s}}\to {\hat{\alpha }}_{s}\text{ and }\beta_{A_{s}}\to {\hat{\beta }}_{s}. \end{array}$$

This notation will be used in later sections. The reader should be reminded that the parameters are derived from each subject and hence are different across subjects. Notice that in a prediction exercise (instead of retrospective regression formulated here), the learning can be applied incrementally for each future observation with each update using just the new observation.

### 3.3. Virtual patient profile

When a new patient (denoted by *s* = *) is received, one can measure their baseline variables b_*_, but not their model parameter space ω_*_. The goal is to estimate a *virtual patient profile* that is believed to best describe the new patient using only the available baseline measurements. To do this one derives the mathematical mapping functions from the baseline characteristics of a patient to their posterior parameters b_*_ ↦ Pr(ω_*_) and $\mathrm{Pr}\left(\sigma_{*}^{2}\right)$, such that a prediction can be made from the baseline variables. These functions are forged using machine learning on the existing pool of training data. Since Pr(ω_*s*_) is parameterized by $\left(\tau_{s},\Lambda_{s}^{-1}\right)$ and likewise $\mathrm{Pr}\left(\sigma_{s}^{2}\right)$ by (α_*s*_, β_*s*_), one has to learn the mappings from the baseline variables to the parameters. The learnt mathematical mapping functions can then be used to obtain estimates of $\left({\hat{\tau }}_{*},{\hat{\Lambda }}_{*}^{-1}\right)$ and $\left({\hat{\alpha }}_{*},{\hat{\beta }}_{*}\right)$, which represent the virtual patent profile for the new patient in the model space.

Due to the conjugate nature of the priors, one does not need to derive the hyperparameters ${\hat{\alpha }}_{*}$ and ${\hat{\beta }}_{*}$ from Equation (5) for the purpose of having point estimates for the treatment response prediction. However, these hyperparameters are necessary in order to derive the posterior distribution of the predicted value—commonly referred to as the posterior predictive distribution. Knowing the distribution allows us to approximate the uncertainties of the estimates, e.g., 95% confidence intervals. Two methods were proposed for learning the mappings from the baseline variables to the virtual patient profile and they are introduced in the following subsections.

#### 3.3.1. Method 1: generalized linear regression

To determine the mappings, one finds the functions: a) ${\text{f}}_{\tau }\left({\text{b}}_{*}\right)\approx {\hat{\tau }}_{*}$ and b) ${\text{f}}_{\text{u}}\left({\text{b}}_{*}\right)\approx {\hat{\Lambda }}_{*}^{-1}$ where **u**_*s*_ is a row vector containing non-zero elements of the upper (or lower) triangular part of ${\hat{\Lambda }}_{s}^{-1}$. Since ${\hat{\Lambda }}^{-1}$ is a covariance matrix (hence symmetric), knowledge of the lower/upper half of the off-diagonal elements plus the diagonal elements is sufficient to fully recreate the matrix.

The mappings are learnt from the training data, in which the posterior distributions of ${\hat{\tau }}_{s}$ and ${\hat{\Lambda }}_{s}^{-1}$ are already available through Equation (A5) in Supplementary Material. Linear regression models of the form $\text{Y}=\hat{\text{P}}\text{B}$ were used to model the two mappings, where the matrix **B** has rows of **b**_*s*_ vectors—one for each subject in the training set—and similarly **Y** is composed of rows of a) τ_*s*_ for determining *f*_τ_ or b) **u**_*s*_ for determining *f*_**u**_. The least squares solutions for the models are given by the Moore-Penrose pseudo-inverse,

(6) $$\begin{array}{r} \hat{\text{Q}}={\left({\text{B}}^{⊺}\text{B}\right)}^{-1}{\text{B}}^{⊺}\text{Y}. \end{array}$$

For prediction, *f*_τ_ and *f*_**u**_ can both be formulated as $\text{f}\left({\text{b}}_{*}\right)={\text{b}}_{*}\hat{\text{Q}}$.

The posterior estimates for the hyperparameters for a new patient are taken as the averaged values of ${\hat{\alpha }}_{s}$ and ${\hat{\beta }}_{s}$ across all subjects in the training set, resulting in ${\hat{\alpha }}_{*}=5.5$ and ${\hat{\beta }}_{*}=1.7$.

#### 3.3.2. Method 2: Gaussian kernel weighted averaging

An alternative method is to find ${\hat{\tau }}_{*}$ and ${\hat{\Lambda }}_{*}^{-1}$ using a weighted average of ${\hat{\tau }}_{s^{\prime }}$ and ${\hat{\Lambda }}_{s^{\prime }}^{-1}$, with $s^{\prime }\in {𝕊}_{*}$ being a subset of subjects in the existing training pool whose baseline variables (${\text{b}}_{s^{\prime }}$) were “similar” to those of the new patient (b_*_). Highly similar subjects will have a higher influence on the value of ${\hat{\tau }}_{*}$ and ${\hat{\Lambda }}_{*}^{-1}$. The “(dis)similarity” *d*_*s*_ is measured using the pairwise euclidean distance between **b**_*s*_ and b_*_, such that

(7) $$\begin{array}{r} d_{s}=\left({\text{b}}_{*}-{\text{b}}_{s}\right){\left({\text{b}}_{*}-{\text{b}}_{s}\right)}^{⊺}. \end{array}$$

This is then sorted and the 17.5% of subjects in 𝕊_*_ with the smallest “dissimilarity” values are kept; this percentage value was chosen as it resulted in the lowest validation error. The weighting *w*_*s*_ was taken as the normalized Gaussian kernel

(8) $$\begin{array}{r} w_{s}=\frac{\exp\left(-\lambda \cdot d_{s}\right)}{\underset{\forall s^{\prime }\in {𝕊}_{*}}{\sum }\exp\left(-\lambda \cdot d_{s^{\prime }}\right)} \end{array}$$

where the parameter value λ = 1.15 was chosen as it again resulted in the lowest validation error. Using Equations (7) and (8), one can estimate ${\hat{\tau }}_{*}$ and ${\hat{\Lambda }}_{*}^{-1}$ as

(9a) $$\begin{array}{rc} {\hat{\tau }}_{*} & =\underset{\forall s\in {𝕊}_{*}}{\sum }w_{s}\tau_{s}, \end{array}$$

(9b) $$\begin{array}{rc} {\hat{\Lambda }}_{*}^{-1} & =\underset{\forall s\in {𝕊}_{*}}{\sum }w_{s}\Lambda_{s}^{-1}, \end{array}$$

and similarly the estimates of the hyperparameters are calculated using

(9c) $$\begin{array}{rc} {\hat{\alpha }}_{*} & =\underset{\forall s\in {𝕊}_{*}}{\sum }w_{s}\alpha_{s}, \end{array}$$

(9d) $$\begin{array}{rc} {\hat{\beta }}_{*} & =\underset{\forall s\in {𝕊}_{*}}{\sum }w_{s}\beta_{s}. \end{array}$$

### 3.4. Prediction using the posterior predictive distribution

When a new subject visits the clinician, their b_*_ vector may be measured and used to approximate ${\hat{\tau }}_{*}$, ${\hat{\Lambda }}_{*}^{-1}$, ${\hat{\alpha }}_{*}$ and ${\hat{\beta }}_{*}$ using either of the methods in the previous subsections. Given a hypothetical medication input x_*_, the treatment response for the new subject can then be predicted through the posterior predictive distribution

(10) $$\begin{array}{r} \mathrm{Pr}\left(y_{*}\right)=t_{\nu }\left({\text{x}}_{*}{\hat{\tau }}_{*},\frac{{\hat{\beta }}_{*}}{{\hat{\alpha }}_{*}}\left(\text{I}+{\text{x}}_{*}{\hat{\Lambda }}_{*}{\text{x}}_{*}^{⊺}\right)\right) \end{array}$$

where the number of degrees of freedom for the Student's *t*-distribution is given by $\nu =2{\hat{\alpha }}_{*}$.

Equation (10) may be used to predict the treatment response for this new patient over their course of the treatment directly without learning; that is to treat each appointment as independent and the parameters are not updated. On the other hand, it is possible to perform incremental Bayesian learning over the course of treatment, by using Equation (A5) in Supplementary Material to update the parameters given the treatment outcome measured for each new visit and the associated inputs. Through incremental learning, the model corrects for discrepancies between the true profile and the virtual patient profile of the new patient. As such, one would expect the prediction to improve as data from more visits to the clinic become available. The implementation of these methods is discussed in more detail in Section 4.4.

At some point, the profile of the new patient in terms of the treatment outcome, inputs, and baseline characteristics can be added to the pool of existing patient profiles (training set) to improve the model's generalizability for future patients.

### 3.5. Training and validation

As discussed in Section 3.1, 157 patients with longitudinal data were randomized and 10-fold cross-validation partitions were constructed resulting in 10-folds of training-validation data partitions.

First, for each fold, the framework detailed in Section 3.2 was followed and Bayesian linear regression was performed to fit patient-specific parameters to each patient in the training dataset. Second, either of the methods specified in Section 3.3 was used in order to construct virtual patient profiles for each patient in the validation set, using the patient-specific parameters. Finally, the procedure outlined in Section 3.4 was followed in order to obtain a prediction for patients in the validation set; effectively treating each patient as new.

### 3.6. Dichotomous remission prediction

Although the model was initially formulated to predict a continuous scale of symptom scores, one can explore dichotomizing the outcome into patients who have shown reduced symptoms and those who have not. The justification is that clinicians and doctors are less likely to be interested in a predicted SNAP-IV score or symptom severity scale as opposed to a simple “yes/no” answer as to whether the patient will be in remission for a given medication. A simple way to adapt the current model to do this is to apply a threshold to the continuous symptom score prediction, below which the patient is predicted to be in remission.

Some of the literature loosely defines remission in ADHD as having a large majority of SNAP-IV responses rated in category 0 (not at all) or 1 (a little) (Hechtman, 2005; Chou et al., 2012). Therefore in this paper, the thresholds were chosen such that the approximate continuous symptom score corresponds to the raw responses from the 18-item SNAP-IV questionnaire all lying in category 1. The resulting symptom score thresholds are only slightly different for inattentiveness and hyperactivity (−0.97 vs. −0.92, see Section 2.3).

Using these thresholds, it was found that the proportion of visits when measurements were taken indicates that remission was relatively rare, 160 out of a total of 1, 147 (13.95%) for the inattentiveness score and 139 (12.1%) for the hyperactivity score. This is expected, as forced titration initially starts with a low medication dosage and one would not expect an effective reduction in symptom ratings to remission levels before the dosage was ramped up in later appointments; in addition, because of medical persistence issues patients can drop out before the clinicians are able to find an effective dose.

Note that all the methods in this paper were first used to predict the continuous symptom scores by regressing the baseline variables, medication prescribed to the treatment response at following appointments. Dichotomized remission prediction only occurs at a later stage. Right censoring (where patients prematurely drop out of dose optimization stage without achieving remission) is therefore not an issue; repression methods can utilize the remaining appointment information to model treatment response regardless of whether remission was achieved or not.

## 4. Performance metrics

To facilitate a comparison between the performance of the different approaches, several performance metrics were used. For the regression tasks, one is interested in the deviation in the predicted symptom scores against the true symptom scores; whereas for the remission classification tasks, one is interested in the performance of the classifiers with regard to the probabilities or ratios of true positive, false positive, true negative and false negative cases.

### 4.1. Regression task

The root mean squared (rms) error measure is defined as the square of the averaged squared error across the 10-folds, across subjects and across all appointments for each individual, i.e.,

(11) $$\begin{array}{r} \sqrt{\text{rms}=\frac{1}{10|𝕊|}\overset{10}{\underset{k=1}{\sum }}\underset{\forall s\in 𝕊}{\sum }\overset{A_{s}}{\underset{a=1}{\sum }}A_{s}^{-1}{{|y}_{s}-{ŷ}_{s}|}^{2}} \end{array}$$

where 𝕊 is the set of all subjects considered (e.g., those in the validation set), |𝕊| denotes the number of subjects in 𝕊; *y*_*s*_, ŷ_*s*_, and *A*_*s*_ are, respectively, the true outcome symptom score, the fitted or predicted outcome symptom score, and the total number of appointments for the individual subject *s*.

### 4.2. Classification task

#### 4.2.1. Sensitivity and specificity

The sensitivity (SEN, also known as the true positive rate or recall) is defined as *N*_TP_/*N*_P_ where *N*_TP_ is the number of *true positives*—appointments where measurements indicated remission and were correctly predicted as such; and *N*_P_ is the actual number of positive cases, i.e., the number of appointments where the corresponding subjects were indeed in remission. This is reported in addition to the specificity (SPC, also known as the true negative rate or fall-out), defined as *N*_TN_/*N*_N_, where *N*_*TN*_ is the number of *true negatives*—those *not* in remission and correctly predicted as such; and *N*_*N*_ is the actual number of negative cases (Fletcher and Fletcher, 2005). Note that if one lets *N*_FP_ and *N*_FN_ be the number of false positives and false negatives respectively, then *N*_P_ = *N*_TP_ + *N*_FN_ and *N*_N_ = *N*_TN_ + *N*_FP_ (Fletcher and Fletcher, 2005).

Sensitivity characterizes the ability of a classifier to rule out false negative predictions (type-II errors) given that a condition is true. On the other hand, specificity measures the ability of a classifier to rule out false positive predictions (type-I errors) given that a condition is false. In this exercise, the sensitivity measure is more important; due to the rarity of remission, and the goal is to try to predict what level of medication is required to achieve remission, the ability of a classifier to recall remission cases (ruling out type-II errors) is more important than ruling out type-I errors.

#### 4.2.2. PPV and NPV

The positive predictive value (PPV, also known as the precision) is the proportion of true positives in the *predicted* positive cases and is the probability of remission given a positive prediction by the algorithm. As such, the PPV is a measure of the “quality” of a given positive prediction. PPV is given by *N*_TP_/(*N*_TP_ + *N*FP). Conversely, the negative predictive value (NPV) is the proportion of true negatives in the *predicted* negative cases, and is the probability of non-remission given a negative prediction. NPV is given by *N*_TN_/(*N*_TN_ + *N*FN) (Fletcher and Fletcher, 2005).

By the argument outlined above, the PPV is more important for this exercise than the NPV.

#### 4.2.3. Balanced accuracy

The overall accuracy of a dichotomous predictor is defined by

$$\text{Accuracy}=\left(N_{\text{TP}}+N_{\text{TN}}\right)/N,$$

where *N* = 1, 147 is the total number of appointments across all subjects.

However, the overall accuracy measure is known to be problematic when the prevalence of success/failure is low (Alberg et al., 2004), i.e., the data are imbalanced (see also the end of Section 3.6). Due to this, some of the literature uses the balance accuracy (BAC) measure, defined as the average of sensitivity and specificity (Brodersen et al., 2010). This is the accuracy measure used throughout this paper. Note that, numerically, the BAC is closely related to the Youden's *J*-statistic (Youden, 1950), also known as “informedness” or “DeltaP′” (Powers, 2011), since it is equal to sensitivity plus specificity minus one.

#### 4.2.4. ROC and AUC

The receiver operating characteristic (ROC) curve is commonly used in the medical and the machine learning community to evaluate the performance of binary classifiers Fawcett (2006). It plots the true positive rate (sensitivity) against the false positive rate (one minus specificity) for a given classifier. A curve is obtained when its classification performance can be tuned through setting a threshold or changing a parameter, trading off the true positive rate against the false positive rate. Binary classifiers that can achieve good compromise between sensitivity and specificity have a large area-under-the-curve (AUC), and this single metric may be used to compare the performance between the different classifiers Bradley (1997).

### 4.3. Trading off sensitivity and specificity

From Section 3.6, the proportion of appointments without remission is (100−13.95)% = 86.05%. Therefore, given this statistic, one would expect that a null model guessing the result randomly would have a sensitivity of 13.95% and a specificity of 86.05%. Simply using point estimates of the continuous symptom score from the learning in the model space approach and thresholding them to give dichotomous predictions of remission results in classifiers with low sensitivity values between 22 and 28% and high specificity values of 94–97%. Due to the low number of remission cases compared to the non-remission cases, the classification is biased against predicting the remission cases, leading to low sensitivity (but high specificity). A classifier can be tuned to improve its sensitivity performance by trading off specificity to a certain degree. A good compromise would be maximizing both sensitivity and specificity equally, which is in essence maximizing the BAC or the Youden's *J*-statistic in Section 4.2.3.

**Figure 5:**
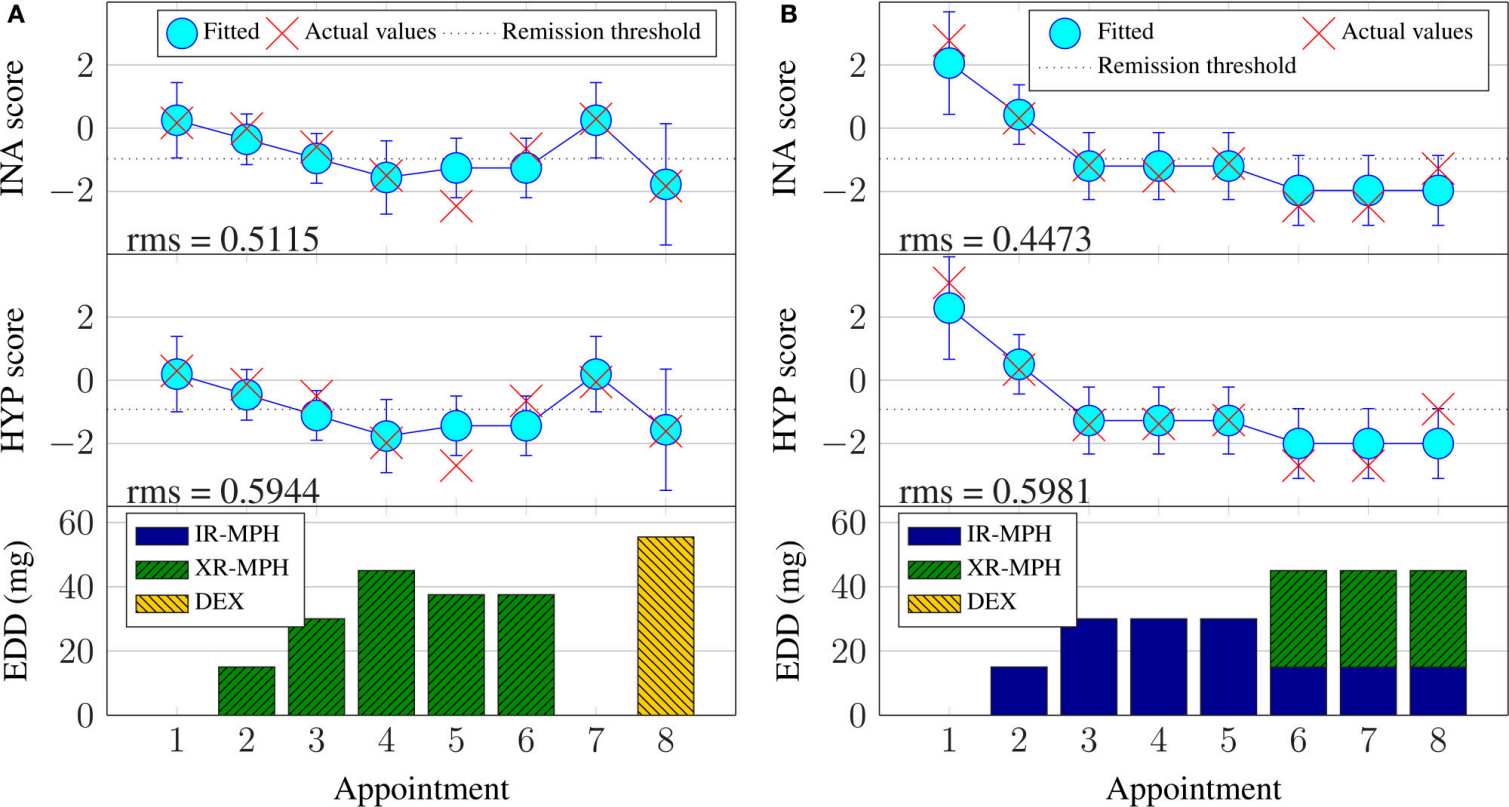
**Examples of Bayesian linear regression on continuous inattentiveness (INA) and hyperactivity (HYP) symptom scores with the Bayesian linear regression training dataset (**BRR**)**. **(A)** Subject #11, **(B)** Subject #74.

**Figure 6:**
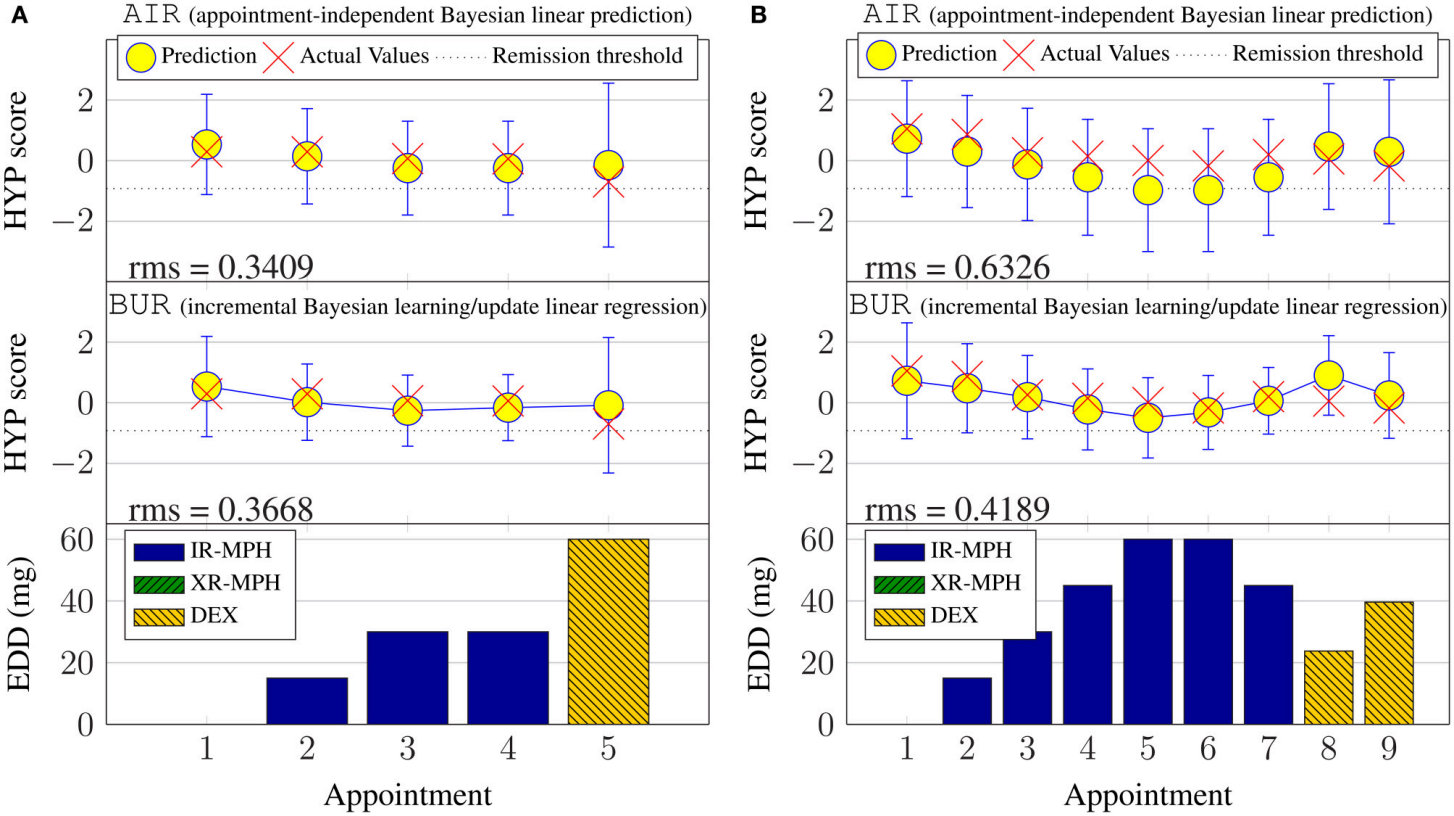
**Examples of continuous hyperactivity symptom score prediction with the validation set**. **(A)** Subject #6. **(B)** Subject #148.

The thresholds for remission are defined by the SNAP-IV symptom factor scores as in Section 2.3, and this defines the ground truth of whether a patient is in remission or not. However, one can take advantage of the fact that the predicted continuous symptom scores from the learning in the model space approach form full posterior distributions with uncertainties associated, and the levels of uncertainty are known (e.g., see the error bars in Figures 5, 6). One may define a critical value as the lower bound of the prediction, above which the probability of the prediction being correct is *x%*. Instead of the remission thresholds comparing against the point estimates, they may be compared against the point estimates minus a critical value. The larger the critical value, the higher the prediction score has to be in order to be classified as not in remission. This in effect is equivalent to raising the threshold, classifying more and more cases into remission, which increases the sensitivity and lowers the specificity. The range of “thresholds” or classifier parameter settings that makes this trade-off can be used to generate a ROC plot (Section 4.2.4). A similar trade-off can be made with classical machine learning algorithms and will be discussed in Section5.

The training data are used to find an optimal classifier setting in order to achieve the highest BAC, and the same classifier setting is then used to classify the validation data. This ensures that the validation data are not used to minimize the validation error.

### 4.4. Benchmarking and implementation

The Bayesian learning in the model space approach relies on prior knowledge (Section 3.2.1) and virtual patient profiles in the model space (Section 3.3), as well as iterative learning (Bayesian update) in order to function. To assess whether these components contribute to the prediction capability of the model, several implementation strategies are investigated, namely:

1. **Appointment-independent prediction (AI):** Treating each appointment as independent (as the first appointment) and giving a prediction only using the virtual patient profile;
2. **Incremental Bayesian linear regression (BR):** The first prediction is performed in exactly the same manner as the appointment-independent case. Then, Bayesian linear regression using elicited priors (see Section 3.2.1) is applied progressively. That is, the effect/outcome of medication prescribed in appointment 1, then observed at appointment 2, is used in the regression model. Then, at appointment 3, outcomes from appointments 1 and 2 are used. Similarly, at appointment 4, outcomes from appointments 1, 2 and 3 are used, and so on. This means that except for the first prediction, the incremental Bayesian linear regression learns from scratch the patient-specific parameters (at each appointment) using the elicited priors. This essentially disregards any information already learnt from the current training set (the virtual patient profiles), treats the validation set as a new “training” set, and performs basic Bayesian linear regression fitting. However, instead of all appointment outcomes being available for each new patient, as is the case during the training phase, one simulates the fact that information is progressively collected during the course of treatment for new patients. Since the virtual patient profiles are not utilized, this serves as a benchmark reference to evaluate the effectiveness of the constructed virtual patient profiles when compared with the next case; this represents a method that can be implemented even when no training data exist.
3. **Incremental Bayesian learning/update (BU):** The first appointment is predicted as for the previous two cases, but then when the true value is observed (in appointment 2), it is fed-back into the Bayesian learning model, i.e., Equation (A5) in Supplementary Material. This updated model is then used to generate a prediction. This progressive updating continues up to the most recent appointment. The crucial difference between this method and the BR method is that, here, the priors used were derived from the virtual patient profiles, as opposed to the elicited priors used in the BR method. When compared to the BR case, this highlights whether the model space offers any utility in aiding the prediction of treatment response.

The Bayesian approach was implemented *ad hoc* in MATLAB software with custom routines. The performance of the prediction was compared across the three different implementations above, using the validation data.

## 5. Comparison with conventional methods

To provide context to the results achieved using the learning in the model space approach, the performance of conventional linear regression methods and machine learning methods was also investigated.

### 5.1. Mixed effects models

Linear mixed effects models (MEM) are widely used in many fields; for instance, biology (Rico et al., 2007), ecology (Stevens et al., 2007), linguistics (Nooteboom and Quené, 2008) and social sciences (Kliegl et al., 2009). They extend upon classical linear regression techniques to support data that have some form of grouping. For example, in this paper, each patient had one or more clinical appointments, and the data from each subject form a group. For each patient *s* with a number of appointments (from 1 to *A*_*s*_), the severity symptom score vector **y**_*s*_ (as in Equation 2) is, for simplicity, assumed to have a linear relationship with the baseline and treatment effect via the following formulation:

(12) $$\begin{array}{r} {\text{y}}_{s}=q_{s}+b_{0}+{\hat{\text{X}}}_{s}\omega_{s}\;+\;\epsilon , \end{array}$$

where ${\hat{\text{X}}}_{s}$ is similar in structure to **X** defined in Equation (4) but without the last column of ones; ω_*s*_ ∈ ℝ_*P*×1_ is the subject-specific parameter vector for the fixed effects, $q_{s}\sim N\left(0,\sigma_{q}^{2}\right)$ is the random effect affecting only the intercept *b*_0_, and $\epsilon \sim N\left(0,\sigma_{\epsilon ,s}^{2}\right)\in {\mathbb{R}}_{A_{s}\times 1}$ is an error term assumed to have a normal distribution. Observe that in this model, the scalar intercept *b*_0_ is a fixed component for all of the patients in the population and the random effect *a*_*s*_ is a subject-specific scalar and is not grouped under any other parameters.

A linear mixed effects model was constructed within the R software (R Core Team, 2013), using the package “*lme4*” (Bates et al., 2015). Severity score predictions were produced by performing out-of-sample forecasts, i.e., on the validation data for each of the folds, using the “*predict*” function in the R software. To generate a prediction, the random effects are assumed to be zero and the population intercept was used. The continuous symptom score regression results for the MEM are prefixed MER.

For a dichotomized clinical remission classification, the symptom score thresholds 0.92 and 0.97 from Section 2.3 for hyperactivity and inattentiveness were used to generate the ground truths. Following the rationale in Section 4.3, the symptom scores predicted by the MEM are given thresholds at different levels to produce a set of classifiers trading off sensitivity against specificity. These threshold-adjusted classifiers are labeled taMEC. The best (in terms of Youden's statistic) threshold settings found using the training data were used for the validation data; the thresholds were 0.05 and -0.20, respectively, for the inattentiveness and hyperactivity symptom scores. In addition, the “*melogit*” function in the Stata software (StataCorp, 2015) was used to directly estimate a mixed effects logistic regression model—a MEM with a logistic link function that predicts the probability of the binary remission outcome. In this case, the threshold procedure was applied to the probabilities rather than the raw symptom scores. The resulting classifier is labeled lrMEC.

### 5.2. Support vector machines and Gaussian processes

In addition to MEM, machine learning classification approaches using support vector machines (SVM) and Gaussian processes (GP) were benchmarked. Both the SVM and GP learning methods are kernel machines and were implemented using linear (dot product kernel: *k*(**x**_*i*_, **x**_*j*_) = (**x**_*i*_.**x**_*j*_)) and nonlinear kernels (the Gaussian kernel: $\text{k}\left({\text{x}}_{i},{\text{x}}_{j}\right)=\exp\left[-\gamma {\left(∥{\text{x}}_{i}-{\text{x}}_{j}∥\right)}^{2}\right]$). Readers are invited to refer to Burges (1998) for a detailed description of support vector machines and to Rasmussen and Nickisch (2010) for a detailed description of GP learning. Compared to MEM and the learning in the model space approach, the SVM and GP are non-parametric methods—there are no subject specific parameters to identify; the models map the subject-specific inputs, such as baseline characteristics and the medication dosage, to the output symptom scores.

For the SVM, nested cross-validation was employed to optimize the parameters in the model. A broad log range spanning [10^−3^:10^2^] was arbitrarily chosen as the search range for the regularization parameter *C*. Similarly, the gamma parameter of the Gaussian kernel was optimized in the log range spanning [10^−4^:10^1^]. For the GP, the model parameters were optimized using conjugate gradient descent, avoiding the need for nested cross-validation. SVM and GP learning approaches were employed as regression models (support vector regression SVR and Gaussian process regression GPR) for the linear and nonlinear kernels to predict the clinical scores. Dichotomous remission predictions were obtained by thresholding the distance from the hyperplane for the SVM, and for thresholding the probabilistic predictions of class membership for GP. These binary classifiers are respectively labeled as SVC and GPC.

From Section 3.6, the number of remission cases outweighed non-remission cases by a ratio of roughly 1:7. For a classification task, this imbalance of data is problematic for many classification algorithms (He and Garcia, 2009). To help alleviate this, a downsampling approach was implemented for both linear and nonlinear kernels of the SVM and GP classifiers dsSVC and dsGPC. During the training phase, the non-responder class was downsampled randomly to match the number of training instances in the remission class. By repeating this downsampling procedure, an ensemble of 1,000 classifiers was trained. A classification prediction was generated by majority voting of the ensemble. Additionally, for the SVMs, an alternative is to learn the regularization parameters *C* on a per-class basis. The rationale is that a higher penalty for errors can be placed on the more abundant class (Osuna et al., 1997); this method is referred to as the weighted SVM (rwSVC). The per-class *C* parameters *C*^+^ and *C*^−^ were optimized using the ranges [10^−3^:10^1^] and [10^−2^:10^2^], respectively. Finally, for the Gaussian process classifier, it is possible to calibrate the probabilistic predictions in order to help account for imbalanced data (Bishop, 2006); this approach is referred to as a re-calibrated GP (rcGPC).

Using Matlab software, the SVM was implemented using the libsvm toolbox (Chang and Lin, 2011), the Gaussian process learning was implemented using the GPML toolbox (Rasmussen and Nickisch, 2010).

## 6. Results and discussions

### 6.1. Continuous symptom score prediction

The rms errors across all models are reported in Table 1.

Table 1**Rms errors for predicting symptom scores for inattentiveness (INA) and hyperactivity (HYP) using the (A) learning in model space and (B) conventional approaches**.

**Table:** 

**(1A) Learning in model space**				
	**Inattentiveness**		**Hyperactivity**	
	**Method 1**	**Method 2**	**Method 1**	**Method 2**
AIR^*^	0.98	0.84	0.97	0.85
BRR^†^	0.82	0.82	0.84	0.84
BUR^‡^	0.99	0.73	1.01	0.75

* AIR*: appointment-independent Bayesian linear prediction*.

† BRR*: retrospective Bayesian linear regression*.

‡ BUR*: incremental Bayesian learning/update linear regression*.

**Table:** 

**(1B) Conventional approaches**				
**Kernel**	**Inattentiveness**		**Hyperactivity**	
	**Linear**	**Nonlinear**	**Linear**	**Non-linear**
SVR^*^	0.73	0.74	0.76	0.81
GPR^†^	0.72	0.77	0.76	0.84
MER^‡^	0.82		0.83	

* SVR*: support vector machine regression*.

† GPR*: Gaussian processes regression*.

‡ MER*: mixed effects regression*.

#### 6.1.1. Learning in the model space approach

Looking at the learning in the model space approach, one can observe that the virtual patient profile construction method, labeled Method 2, resulted in lower errors overall compared to Method 1. In Method 1, the mappings were learnt using simple linear regression from baseline variables to the parameter space. In addition to this simple linear regression, low degree polynomial (quadratic to quartic) basis functions were tried; whilst degrees up to a cubic resulted in a slightly lower training error, there was worse generalizability (i.e., higher validation error). For Method 2, the incremental Bayesian learning (BUR) approach performed the best overall; its performance advantage over the appointment-independent prediction (AIR) approach is expected given that it allows the model to adapt to a new patient as the treatment continues. The performance advantage over the retrospective Bayesian linear regression (BRR) approach can be attributed to the fact that the virtual patient profile (Section 3.3) had utilized the prior whilst the Bayesian linear regression only uses the elicited prior (see Section 3.2.1). This supports the fact that the training population was able to add valuable information to the prediction task.

We recall that the BUR constructs virtual patient profiles while the BRR only uses the prior knowledge. It is interesting to note that the BRR outperforms the BUR using Method 1, suggesting that Method 1 was not an effective method for incorporating information from existing patient models.

**Figure 7:**
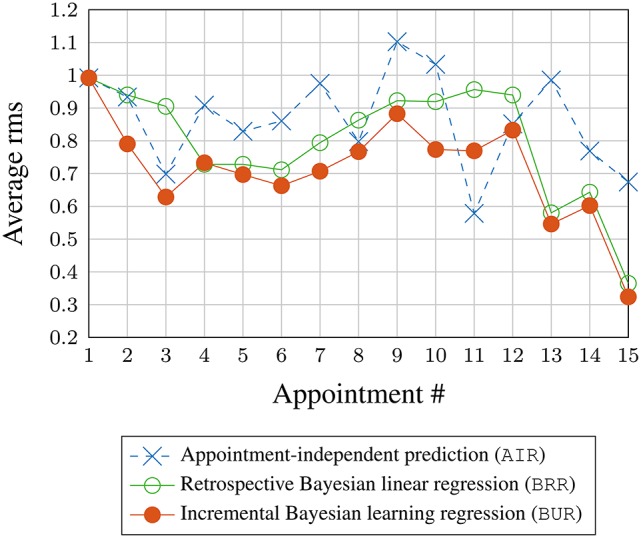
**Rms prediction (validation) error averaged across all subjects vs. appointment number**.

Figure 7 shows the rms values averaged across all subjects during the validation phase and sorted by the clinical appointment (visit) number. Data above 15 visits are not shown as only a single patient had more than 15 visits. There is a slight downward trend visible with the BUR; suggesting that incremental Bayesian learning approach is able to reduce the prediction error as more data are known about a new patient through repeated appointments. The BRR also shows a downward trend, but the error is slightly higher than the BUR. This is because the BRR starts with only the elicited prior and performs learning (fitting) when more data are available, unlike the BUR which starts off with information from the training set in the form of a constructed/estimated virtual patient profile.

Figure 5 illustrates some examples of Bayesian linear regression performed during the training phase and their associated fitting rms errors. It can be seen that Bayesian linear regression fits similarly well for both the inattentiveness and hyperactivity symptom scores. Switching to a new type of medicine is usually associated with larger uncertainty (error bars). Looking at subject #74 (Figure 5B) in particular, it can be seen that, despite having the same input dosage from appointments 6–8, there were variations in the severity of the ADHD symptoms. It is not possible to know the exact reason for the variation for this subject during this particular period, without further information—perhaps this was due to adherence issues (the patient not taking their medication as prescribed), physiological factors, measurement “noise,” or perhaps something else entirely. By design, Bayesian linear regression can only fit the same outcome given the same input. This does highlight the fact that the current model may not have enough information in the form of covariates to account for some of these factors.

A subset of results for the validation phase is plotted in Figure 6. For brevity, only prediction outcomes for hyperactivity symptom scores are shown and retrospective Bayesian linear regression results (BRR) are omitted. The lack of solid lines connecting the predictions in the topmost subplot serves as a reminder that the model does not incorporate temporal aspects for the case of appointment-independent (AIR) prediction, which treats each appointment as the first (new) appointment for a new patient. This is also why the 95% confidence intervals for AIR are larger (more uncertain) than those for the BUR. Also note that, by design, the prediction results for the first appointment are identical for both approaches.

The figures illustrate that, during prognosis, incremental learning does not always improve the prediction error compared to simply predicting at every appointment without updating the model using new information. However, based on the rms errors in Table 1A, one expects incremental learning to perform better overall across subjects, especially for subjects with a prediction offset, such as over- or under-estimates. This is illustrated by the results for subject #148 given in Figure 6B, where the virtual patient profile for this patient consistently underestimates the actual hyperactivity score. Here, the incremental Bayesian learning was able to adapt the parameter ω and shifted the prediction upwards, resulting in lower prediction errors over the subsequent appointments.

#### 6.1.2. Conventional machine learning approaches

Looking at the results in Table 1B, the conventional approaches yield similar performance, with linear SVR and GPR methods performing better than their nonlinear counterparts. The mixed effects model has slightly worse results. Errors of linear SCR and linear GPR are similar to each other, and to those for the learning in the model space approach BUR with Method 2. We conclude that for the task of predicting continuous symptom scores with the dataset investigated, the learning in the model space approach performs comparably with conventional approaches.

### 6.2. Dichotomous remission prediction

#### 6.2.1. Learning in the model space approach

**Figure 8:**
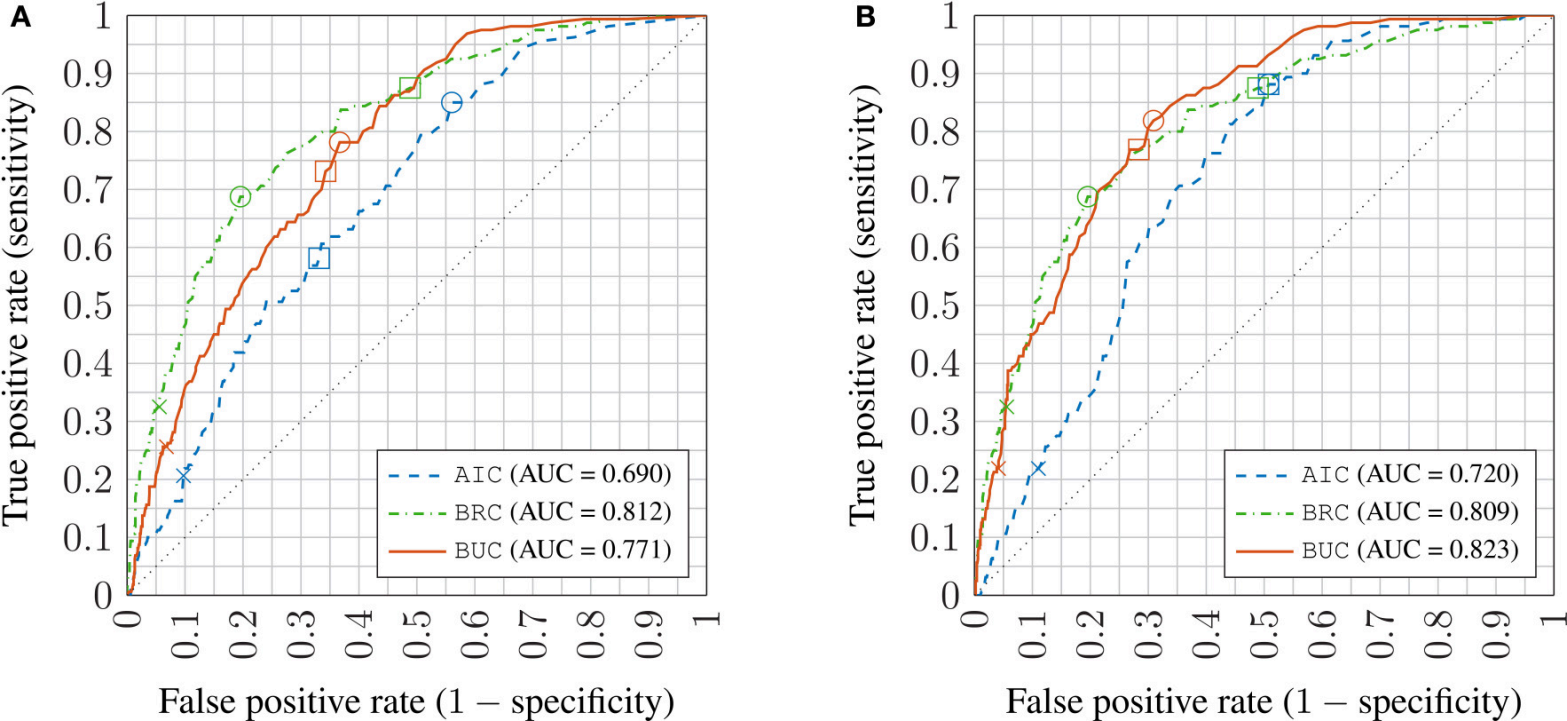
**Receiver operator characteristic (ROC) plots of inattentiveness prediction using virtual patient profile constructed by Methods 1 and 2 (A,B)** in the learning in model space approach; crosses: no critical value adjustment (based on point estimates); squares: best performing critical values on training set; circles: best performing critical values on the validation set; AUC: Area under the ROC curve AIC: appointment-independent classifier; BRC: retrospective Bayesian linear regression classifier; BUC: incremental Bayesian learning/update classifier.

For the learning in the model space approach, the ROC curves for the dichotomous predictor are plotted in Figure 8 for both of the virtual patient profile (Section 3.3) construction methods. As the results for inattentiveness and hyperactivity scores were similar, only the ROC curves for inattentiveness are shown. The AUC values are given in the legend. The crosses on the lines mark the resulting classifier performance if one uses point estimates for the continuous symptom score from the model and simply applies the clinical remission thresholds. The squares mark the classifiers that have critical values based on maximizing the Youden's *J*-statistic (or the BAC, see Section 4.2.3) for the training set—this is equivalent to the sensitivity and specificity measures being maximized equally as a function of the critical values. Lastly, the circles mark the best classifier for the validation set in terms of the Youden's *J*-statistic. The closer the squares are to the circles, the better optimized the classifier is assuming no knowledge of the validation dataset. Those optimized classifiers marked by squares in the graph are used to generate various binary classifier performance metrics (see Section 4.2) in Table 2.

**Table 2:** **Sensitivity, specificity, accuracy, and AUC of the remission classifier with critical values adjusted with respect to uncertainties in the predicted symptom scores**.

		**Inattentiveness**				**Hyperactivity**			
		**Method 1**		**Method 2**		**Method 1**		**Method 2**	
CFM^a^	AIC^*^	93	327	141	501	82	314	121	448
		67	660	19	486	57	694	18	560
	BRC^†^	140	482	140	483	125	480	125	483
		20	505	20	504	14	528	14	525
	BUC^‡^	117	338	123	280	97	307	109	253
		43	649	37	707	42	701	30	755
SEN^b^	AIC	58.1%		88.1%		59.0%		87.1%	
	BRC	87.5%		87.5%		89.9%		89.9%	
	BUC	73.1%		76.9%		69.8%		78.4%	
SPC^c^	AIC	66.9%		49.2%		68.9%		55.6%	
	BRC	51.2%		51.1%		52.4%		52.1%	
	BUC	65.8%		71.6%		69.5%		74.9%	
BAC^d^	AIC	62.5%		68.7%		63.9%		71.3%	
	BRC	69.3%		69.3%		71.2%		71.0%	
	BUC	69.4%		74.3%		69.7%		76.7%	
PPV^e^	AIC	22.1%		22.0%		20.7%		21.3%	
	BRC	22.5%		22.5%		20.7%		20.6%	
	BUC	25.7%		30.5%		24.0%		30.1%	
NPV^f^	AIC	90.8%		96.2%		92.4%		96.9%	
	BRC	96.2%		96.2%		97.4%		97.4%	
	BUC	93.8%		95.0%		94.4%		96.2%	
AUC^g^	AIC	69.0%		72.0%		68.0%		73.8%	
	BRC	81.2%		80.9%		83.6%		83.3%	
	BUC	77.1%		82.3%		76.7%		84.4%	

a *CFM: confusion matrix*.

b *SEN: sensitivity*.

c *SPC: specificity*.

d *BAC: balanced accuracy*.

e *PPV: positive predictive value*.

f *NPV: negative predictive value*.

g *AUC: area under ROC curve*.

* AIC*: appointment-independent classifier*.

† BRC*: retrospective Bayesian linear regression classifier*.

‡ BUC*: incremental Bayesian learning/update classifier*.

*Shaded values represent best performance amongst the compared methods*.

Looking at Table 2. The confusion matrices (CFM) show the number of true positives and false negatives in the first column, and false positives and true negatives in the second column. These may be used to calculate any classification performance metrics not included in this paper, such as the *F*-measure.

Similar to the continuous symptom prediction task, the BRC outperforms the AIC showing that posterior information is utilized effectively. As in the continuous task, the virtual patient profile construction method labeled Method 2 is better overall than Method 1, but the difference is much smaller in the classification task and the advantage is not universal across all metrics, especially for the AIC. Note that the virtual patient profile construction method has little effect on the BRC as it does not use it. The BRC achieves higher sensitivity values but a lower PPV compared to the BUC, meaning that the BRC is better at recalling remission cases, but the remission predictions by the BUC are more reliable. The SPC achieved by the BUC is notably higher, being better at ruling out false positives.

#### 6.2.2. Conventional machine learning approaches

Table 3 shows the binary classifier performance metrics for the conventional machine learning approaches. Apart from the AUC, all of the other metrics in the table were derived from classifier settings (set-points) that had optimized the balanced accuracy (BAC) during the training stage. Apart from rcGPC, the BAC values across the different approaches are similar. The MEC classifiers perform well compared with GPC and SVC, with consistently high AUC values for both inattentiveness and hyperactivity. However, the set-points of the MEC classifiers achieve lower sensitivity (but higher specificity) than the SVC. As mentioned in Section 4.2, a higher sensitivity is more important for this exercise. PPV is the other measure of interest; the lrMEC, in particular, achieved the highest PPV amongst all the conventional approaches—partially helped by its low sensitivity.

**Table 3:** **Sensitivity, specificity, accuracy, and AUC of the remission classifier with critical values adjusted with respect to uncertainties in the predicted symptom scores**.

		**Inattentiveness**		**Hyperactivity**	
		**Linear**	**Non-linear**	**Linear**	**Non-linear**
Sensitivity	dsSVC^a^	70.0%	70.6%	67.6%	66.9%
	dsGPC^b^	68.1%	67.5%	67.6%	66.9%
	rwSVC^c^	76.9%	33.8%	76.9%	69.1%
	rcGPC^d^	43.1%	43.2%	71.3%	48.2%
	taMEC^e^	60.0%		58.9%	
	lrMEC^f^	53.1%		56.0%	
Specificity	dsSVC	61.9%	62.3%	67.8%	66.6%
	dsGPC	67.9%	69.1%	67.8%	71.3%
	rwSVC	55.9%	77.6%	62.1%	62.1%
	rcGPC	59.0%	18.4%	50.7%	50.6%
	taMEC	73.3%		73.8%	
	lrMEC	81.4%		81.4%	
Balanced accuracy	dsSVC	66.0%	66.5%	67.7%	66.7%
	dsGPC	68.0%	68.3%	67.7%	69.1%
	rwSVC	66.4%	55.7%	69.5%	65.6%
	rcGPC	51.1%	44.8%	46.9%	49.4%
	taMEC	66.6%		66.4%	
	lrMEC	67.2%		70.6%	
Positive predictive value	dsSVC	23.0%	23.3%	22.4%	21.6%
	dsGPC	25.6%	26.2%	22.4%	24.3%
	rwSVC	22.0%	19.6%	21.9%	20.1%
	rcGPC	15.6%	12.4%	10.8%	11.9%
	taMEC	26.7%		23.7%	
	lrMEC	31.6%		29.0%	
Negative predictive value	dsSVC	92.7%	92.9%	93.8%	93.9%
	dsGPC	92.9%	92.9%	93.8%	93.4%
	rwSVC	93.7%	87.8%	95.1%	93.6%
	rcGPC	86.5%	79.8%	86.6%	87.63%
	taMEC	91.8%		92.9%	
	lrMEC	91.5%		94.1%	
Area under ROC curve	dsSVC	71%	69%	73%	71%
	dsGPC	75%	71%	73%	70%
	rwSVC	71%	60%	76%	71%
	rcGPC	49%	41%	46%	48%
	taMEC	74.8%		77.5%	
	lrMEC	75.8%		77.2%	

a dsSVC: down-sampled support vector machine classifier;

b dsGPC: down-sampled Gaussian processes classifier;

c rwSVC: regularization-weighted support vector machine classifier;

d rwGPC: regularization-weighted support Gaussian processes classifier;

e taMEC: threshold-adjusted mixed effects classifier;

f *lrMEC: logitic regression mixed effects classifier*.

*Shaded values represent best performance amongst the compared methods*.

**Figure 9:**
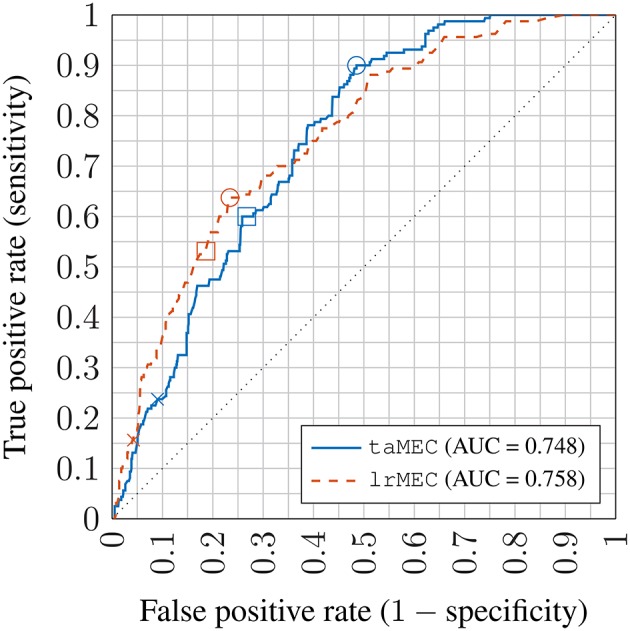
**Receiver operator characteristic (ROC) plot of inattentiveness prediction using mixed effects models; crosses: no threshold adjustment (based on point estimates); squares: best performing threshold setting on training set; circles: best performing threshold setting on the validation set; AUC: Area under the ROC curve; **taMEC**: threshold-adjusted mixed effects classifier; **lrMEC**: logitic regression mixed effects classifier**.

The ROC plot for the MEC is shown in Figure 9. The lrMEC variant fitted the training set better but both the lrMEC and the taMEC achieve similar validation performance. Tracing the ROC values, the lrMEC seems more suitable for high specificity settings while taMEC appears to be more suitable for high sensitivity classification.

For the machine learning approaches GPC and SVC, linear models work better. This was similarly observed in the continuous symptom score prediction task. Comparing methods in tackling data imbalance, the weighted SVM classifier rwSVC method performed better than the downsampled dsSVC method, while the downsampled Gaussian process classifier dsGPC method performed better than the re-calibrated rcGPC. Looking at both the BAC and AUC metrics, rwSVC and dsGPC perform similarly, with the former slightly better at classifying remission of hyperactivity, whereas the latter is slightly better for inattentiveness.

Overall for the conventional methods, the rwSVC achieves the best compromise between SEN and PPV, meaning that it can identify remission cases more readily and at the same time the remission predictions are more reliable. Comparing Tables 2, 3 it can be seen that the learning in the model space approach is superior overall. With respect to the BAC and AUC measures, the best performing BUC approach has an advantage of about 6–7%. This is interesting given the similar performance in the continuous symptom score prediction task amongst all approaches. The rms error measure in the symptom score prediction task was based on point estimate calculations, and thus used no information on the shape of the posterior distribution. The posterior predictive distribution (Section 3.4) for the BUC has a Student's *t*-distribution specific to each patient. The distributions were used to construct a probabilistic threshold in trading off specificity and specificity. This subject-specific nonlinear thresholding procedure may have contributed to its performance advantage over other approaches.

#### 6.2.3. Comparison with literature

As far as the authors are aware, Kim et al. (2015) is the only published literature on treatment response prediction of ADHD patients using machine learning techniques. Their best attempt achieved an AUC value of 0.84 and 86.4% classification accuracy (that is, the percentage of correct predictions, different from the BAC measure used in this paper) using a wide range of information types including demographical, clinical, genetic, environmental, neuropsychological and neuroimaging measures. In comparison, this paper includes only the more readily obtainable demographical and clinical information and is able to achieve best-case AUCs of 0.82–0.84. Restricting to demographical and clinical information, the highest performing method using SVMs in Kim et al. (2015) had an AUC of 0.69. Granted, the comparison is imprecise because the quality, quantity and sources of demographical and clinical information are different between this paper and Kim et al. (2015). Judging from the AUC values achieved by SVMs in this paper of about 0.71 (see Table 3), the results appears to be very close to those in Kim et al. (2015). Due to this similarity, the previous comparisons should be valid.

## 7. Clinical utility and further work

The proposed learning in the model space approach is capable of predicting, for an individual, the minimum dosage of a particular medication required to have a user-defined chance of achieving symptomatic remission. It is highly flexible and potentially can be extended to any disease or disorder where medication is used in the course of treatment, speeding up and reducing the cost of the dose optimization/forced titration process, and potentially improving the quality of life for patients by ending the treatment sooner.

The current model, however, does not take into account adverse drug reactions (ADRs), minimization of which is another goal of a dose optimization titration process. To improve clinical utility, it is essential that ADRs are modeled. While data on this are available from the clinical notes accompaning the ADDUCE trial, a different modeling approach is required to incorporate the many different types of ADRs, with prevalence ranging from infrequent to very rare.

While the proposed approach achieves excellent performance in terms of treatment response classification, there is room for improvement. One obvious way to achieve this is to incorporate more data, especially covariates that are functions of time. In this exercise for example, the body mass index and age variables measured at baseline (first appointment) of the patients contribute to the latent factors, which in turn form the baseline variables. As such, they do not vary over time. It may be worth investigating whether the addition of temporal covariates, such as blood pressure, would improve the model.

Another venue for potential improvement is to extend the linear model to a nonlinear model—there is no guarantee that all the covariates have a linear relationship with treatment response. Identifying the level and nature of nonlinear relationships is the first challenge. In the current Bayesian framework, the introduction of nonlinearities increases computational complexity for Bayesian inference, requiring the use of techniques such as Gibbs sampling.

There are other areas of interest. For example, what is the optimum strategy, in terms of timing and requirements, for incorporating semi-new patient data to the model space to improve the generalizability of the model for other new patients? How can medical adherence/concordance be modeled? Does gender of the patient play a role in their treatment response?

## 8. Conclusion

A learning in the model space framework has been utilized to develop a personalized medicine approach to treatment response prediction. First of all, factor analysis was performed to extract latent factors from a large clinical dataset, collected from a UK sample of 157 patients suffering from attention-deficit hyperactivity disorder. The resulting reduced-order patient information was then encoded in a model parameter space resulting in a cloud of personalized models. Then, the patient-specific model space parameters were used to train a Bayesian linear regression model. New patients are then matched to existing patients most similar to themselves to obtain a virtual patient profile, which in turn forms a prior parameter set for the Bayesian linear regression model. Through a Bayesian update algorithm, new data are continuously integrated to improve the prediction performance for a given patient. In addition, the parameters of the “new” patients can be added to the model parameter space (once sufficient data are available) to improve the generalizability of the model for future patients.

Comparisons were made between the learning in the model space approach with conventional data-driven machine learning and regression approaches. In terms of the prediction of the continuous symptom factor scores, the performance of the learning in model space framework was on a par with conventional approaches. However, the new approach is shown to outperform support vector machines, Gaussian processes and linear mixed effects classifiers in the prediction of symptomatic remission. The effective gain in classification performance of the new model can potentially speed up and reduce the cost of a forced titration or dose optimization titration process, which is normally manually performed by the clinician to assess the effective dosage of medication. Further work includes incorporating the prediction of adverse drug reactions, which is also an important element in the dose optimization titration process.

## Ethics statement

This is secondary data analysis of the ADDUCE ADHD study. ADDUCE has received a favorable ethical opinion from the relevant UK National Health Service (NHS) Research Ethics Committee (REC reference 11/ES/0016). All subjects gave written informed consent in accordance with the Declaration of Helsinki. The protocol was approved by the REC. Consent to information access and secondary data analysis has been granted. All data were handled as per relevant guidelines and rules for such information, including the Data Protection Act 1998 and as per the initial ethical approval.

## Author contributions

HW was responsible for performing feature extraction of the clinical data, literature search, causal factor model simplification, the software implementation of the learning in model space framework, and determined benchmarking protocol. PAT provided the clinical context of ADHD, constructed the prior knowledge used by the Bayesian framework, aided development on the causal factor model, and designed the literature search protocol. PW aided HW on the literature search and written the background on ADHD. OD implemented the SVM and GP machine learning algorithms. BL constructed the linear mixed effects models. YS helped with the technical implementation of the Bayesian linear regression algorithm. PT provided the mathematical formulation of the learning in model space approach and guidance on software implementation. MC and TN suggested candidate modeling approaches compatible with the framework, suggested a number of conventional approaches to be evaluated, appraised the output of the Bayesian approach, and provided recommendations for the improvement of the implementation and the methodology to ensure fair comparisons. SI coordinated access to the clinical data, facilitated digitization of paper-based clinical records, and maintained the database. DC provided the clinical context of ADHD, suggested causal mechanisms for treatment response, and defined the scope of clinical data collection.

## Funding

We gratefully acknowledge the support from the UK Engineering and Physical Sciences Research Council (EPSRC), grant number EP/L000296/1. The ADDUCE project from which this piece of research borrowed clinical data is funded by the European Union's Seventh Framework Programme for research, technological development and demonstration under grant agreement number 260576.

### Conflict of interest statement

The authors declare that the research was conducted in the absence of any commercial or financial relationships that could be construed as a potential conflict of interest.
