# Supplementary material for: Personalized Medication Response Prediction for Attention-Deficit Hyperactivity Disorder: Learning in the Model Space vs. Learning in the Data Space
Source: Front Physiol. 2017 Apr 11;8:199. doi: 10.3389/fphys.2017.00199 (PMC5387107; doi:10.3389/fphys.2017.00199)
Supplement: Supplementary file 1 [file Appendix.pdf]

APPENDIX

Prior distributions and knowledge

With respect to equations (3) and (4), dropping the subscript for subject s for convenience, the outcome likelihood can then be modeled as:

$$\begin{aligned}\Pr\left(\mathbf{y} \mid \mathbf{X}; \boldsymbol{\omega}, \sigma^2\right) &= \mathcal{N}\left(\mu = \mathbf{X}\boldsymbol{\omega}, \sigma^2\mathbf{I}\right) \\ &= \left(\frac{1}{2\pi\sigma^2}\right)^{0.5A_s} \exp\left\{-\frac{1}{2\sigma^2}\left(\mathbf{y}-\mathbf{X}\boldsymbol{\omega}\right)^{\mathsf{T}}\left(\mathbf{y}-\mathbf{X}\boldsymbol{\omega}\right)\right\} \qquad \text{(A.1)}\end{aligned}$$

where $\mathbf{I}$ is the $A_s \times A_s$ identity matrix.

The conjugate prior of the unknowns—the parameter vector $\boldsymbol{\omega}$ and the error variance σ^2 are given by, respectively, $\Pr\left(\boldsymbol{\omega} \mid \sigma^2\right) \sim \mathcal{N}\left(\boldsymbol{\tau}_0, \sigma^2\boldsymbol{\Lambda}_0^{-1}\right)$ and $\Pr\left(\sigma^2\right) \sim \mathcal{G}^{-1}\left(\alpha_0, \beta_0\right)$, where the $\mathcal{G}^{-1}$ is the Inverse-Gamma distribution (O'Hagan and Forester, 2004), such that

$$\Pr\left(\sigma^2\right) = \frac{\beta_0^{\alpha_0}}{\sigma^{2(\alpha_0+1)}\Gamma(\alpha_0)} \exp\left(-\frac{\beta_0}{\sigma^2}\right); \quad \sigma^2, \alpha_0, \beta_0 > 0.$$

Assuming $\boldsymbol{\omega}$ and σ^2 are independent, the joint prior probability density function $\Pr\left(\boldsymbol{\omega}, \sigma^2\right)$ is then given by the Normal-Inverse-Gamma ($\mathcal{N}\mathcal{G}^{-1}$) prior, such that

$$\begin{aligned}\Pr\left(\boldsymbol{\omega}, \sigma^2\right) &= \Pr\left(\boldsymbol{\omega} \mid \sigma^2\right)\Pr\left(\sigma^2\right) = \mathcal{N}\mathcal{G}^{\text{-1}}\left(\boldsymbol{\tau}_0, \boldsymbol{\Lambda}_0^{-1}, \alpha_0, \beta_0\right) \qquad \text{(A.2)} \\ &= \frac{\beta_0^{\alpha_0}}{(2\pi)^{P/2}\left|\boldsymbol{\Lambda}_0^{-1}\right|^{1/2}\Gamma(\alpha)}\left(\frac{1}{\sigma^2}\right)^{\alpha_0+1+P/2} \times \\ &\quad \exp\left\{-\frac{1}{\sigma^2}\left[\beta_0 + \frac{1}{2}\left(\boldsymbol{\omega}-\boldsymbol{\tau}_0\right)^{\mathsf{T}}\boldsymbol{\Lambda}_0^{-1}\left(\boldsymbol{\omega}-\boldsymbol{\tau}_0\right)\right]\right\}. \qquad \text{(A.3)}\end{aligned}$$

Posterior distributions

The posterior distribution can be parameterized as

$$\begin{aligned}\Pr\left(\boldsymbol{\omega}, \sigma^2 \mid \mathbf{y}\right) &\propto \Pr\left(\boldsymbol{\omega} \mid \sigma^2; \mathbf{y}\right)\Pr\left(\sigma^2 \mid \mathbf{y}\right) \\ &= \left(\frac{1}{\sigma^2}\right)^{\alpha_n+1+P/2} \exp\left\{-\frac{1}{\sigma^2}\left[\beta_n + \frac{1}{2}\left(\boldsymbol{\omega}-\boldsymbol{\tau}_n\right)^{\mathsf{T}}\boldsymbol{\Lambda}_n\left(\boldsymbol{\omega}-\boldsymbol{\tau}_n\right)\right]\right\}, \qquad \text{(A.4)}\end{aligned}$$

where the two probabilities on the right-hand side correspond to, respectively, the multivariate normal distribution $\mathcal{N}\left(\boldsymbol{\tau}_n, \sigma^2\boldsymbol{\Lambda}_n^{-1}\right)$ and the inverse-gamma distribution $\mathcal{G}^{-1}\left(\alpha_n, \beta_n\right)$. The parameters of the distributions are obtained through Bayesian learning:

$$\begin{aligned}\boldsymbol{\Lambda}_n &= \mathbf{X}^{\mathsf{T}}\mathbf{X} + \boldsymbol{\Lambda}_0 & \boldsymbol{\tau}_n &= \boldsymbol{\Lambda}_n\left(\boldsymbol{\Lambda}_0\boldsymbol{\tau}_0 + \mathbf{X}^{\mathsf{T}}\mathbf{y}\right) \\ \alpha_n &= \alpha_0 + \frac{n}{2} & \beta_n &= \beta_0 + \frac{1}{2}\left(\mathbf{y}^{\mathsf{T}}\mathbf{y} + \boldsymbol{\tau}_0^{\mathsf{T}}\boldsymbol{\Lambda}_0\boldsymbol{\tau}_0 - \boldsymbol{\tau}_n^{\mathsf{T}}\boldsymbol{\Lambda}_n\boldsymbol{\tau}_n\right) \qquad \text{(A.5)}\end{aligned}$$

where n is the number of observations (O'Hagan and Forester, 2004).

Marginal distributions

The marginal distribution of treatment outcome $\Pr(\mathbf{y})$ is found by integrating out (marginalising) $\boldsymbol{\omega}_*$ and σ^2 over $\Pr\left(\mathbf{y} \mid \boldsymbol{\omega}, \sigma^2\right)$ to obtain $\Pr(y)$. First of all, one computes the distribution $\Pr\left(\mathbf{y} \mid \sigma^2\right)$ by integrating out $\boldsymbol{\omega}$, which is

$$\begin{aligned}\Pr\left(\mathbf{y} \mid \sigma^2\right) &= \int \Pr\left(\mathbf{y} \mid \boldsymbol{\omega}, \sigma^2\right) \Pr\left(\boldsymbol{\omega} \mid \sigma^2\right) \, \mathrm{d}\boldsymbol{\omega} \\ &= \int \mathcal{N}\left(\mathbf{X}\boldsymbol{\omega}, \sigma^2 \mathbf{I}\right) \mathcal{N}\left(\tau_0, \sigma^2 \boldsymbol{\Lambda}_0^{-1}\right) \, \mathrm{d}\boldsymbol{\omega}.\end{aligned}$$

This can be shown to result in

$$\Pr\left(\mathbf{y} \mid \sigma^2\right) = \mathcal{N}\left(\mathbf{X}\boldsymbol{\tau}_0, \sigma^2\left(\mathbf{I} + \mathbf{X}\boldsymbol{\Lambda}_0^{-1}\mathbf{X}^{\mathsf{T}}\right)\right). \tag{A.6}$$

The marginal density $\Pr(\mathbf{y})$ can then be obtained by integrating out a $\mathcal{N}\mathcal{G}^{-1}$ density function, as

$$\begin{aligned}\Pr(\mathbf{y}) &= \int \Pr\left(\mathbf{y} \mid \sigma^2\right) \Pr\left(\sigma^2\right) \, \mathrm{d}\sigma^2 \\ &= \int \mathcal{N}\left(\mathbf{X}\boldsymbol{\tau}_0, \sigma^2\left(\mathbf{I} + \mathbf{X}\boldsymbol{\Lambda}_0^{-1}\mathbf{X}^{\mathsf{T}}\right)\right) \mathcal{G}^{-1}\left(\alpha_0, \beta_0\right) \, \mathrm{d}\sigma^2 \\ &= \int \mathcal{N}\mathcal{G}^{-1}\left(\mathbf{X}\boldsymbol{\tau}_0, \sigma^2\left(\mathbf{I} + \mathbf{X}\boldsymbol{\Lambda}_0^{-1}\mathbf{X}^{\mathsf{T}}\right), \alpha_0, \beta_0\right) \, \mathrm{d}\sigma^2 \\ &= \mathrm{t}_\nu\left(\mathbf{X}\hat{\boldsymbol{\tau}}_s, \frac{\hat{\beta}_s}{\hat{\alpha}_s}\left(\mathbf{I} + \mathbf{X}\hat{\boldsymbol{\Lambda}}_s\mathbf{X}^{\mathsf{T}}\right)\right)\end{aligned} \tag{A.7}$$

where t_ν is the multivariate Student's t-distribution with the number of degrees of freedom $\nu = 2\hat{\alpha}_*$.
